# A Numerical Analysis of the Cooling Performance of a Hybrid Personal Cooling System (HPCS): Effects of Ambient Temperature and Relative Humidity

**DOI:** 10.3390/ijerph17144995

**Published:** 2020-07-11

**Authors:** Pengjun Xu, Zhanxiao Kang, Faming Wang, Udayraj

**Affiliations:** 1 School of Design Art, Xiamen University of Technology, Xiamen 361024, China; xupengjun2012@gmail.com; 2 Thermal Environment and Ergonomics Research Group (TEERG), Institute of Textiles and Clothing, The Hong Kong Polytechnic University, Hung Hom 999007, Hong Kong; zxkang@connect.hku.hk; 3 School of Architecture and Art, Central South University, Changsha 410083, China; 4 Department of Mechanical Engineering, Indian Institute of Technology Bhilai, Chhattisgarh 492015, India; udayraj@iitbhilai.ac.in

**Keywords:** personal cooling system, heat and mass transfer, ambient temperature, relative humidity, phase change materials, thermal management

## Abstract

Hybrid personal cooling systems (HPCS) incorporated with ventilation fans and phase change materials (PCMs) have shown its superior capability for mitigating workers’ heat strain while performing heavy labor work in hot environments. In a previous study, the effects of thermal resistance of insulation pads, and latent heat and melting temperature of PCMs on the HPCS’s thermal performance have been investigated. In addition to the aforementioned factors, environmental conditions, i.e., ambient temperature and relative humidity, also significantly affect the thermal performance of the HPCS. In this paper, a numerical parametric study was performed to investigate the effects of the environmental temperature and relative humidity (RH) on the thermal management of the HPCS. Five levels of air temperature under RH = 50% (i.e., 32, 34, 36, 38 and 40 °C) and four levels of environmental RH at two ambient temperatures of 36 and 40 °C were selected (i.e., RH = 30, 50, 70 and 90%) for the numerical analysis. Results show that high environmental temperatures could accelerate the PCM melting process and thereby weaken the cooling performance of HPCS. In the moderately hot environment (36 °C), HPCS presented good cooling performance with the maximum core temperature at around 37.5 °C during excise when the ambient RH ≤ 70%, whereas good cooling performance could be only seen under RH ≤ 50% in the extremely hot environment (40 °C). Thus, it may be concluded that the maximum environmental RH under which the HPCS exhibiting good cooling performance decreases with an increase in the environmental temperature.

## 1. Introduction

Heatwaves are becoming a significant threat to our society due to global climate change in recent years [1,2]. During heatwave periods, outdoor workers (e.g., in agricultural, industrial or construction sectors) may suffer from rigorous heat strain, which could lead to severe heat-related illnesses and injuries, including heat rashes, heat cramps, heat exhaustion, heatstroke, and even mortality [3,4,5,6]. Recently, due to the outbreak of COVID-19, medical professionals and the frontline health care workforce are required to wear protective masks and clothing for personal health and safety [7,8]. Such personal protective equipment (PPE) could also severely impede body heat dissipation and thereby, uncompensable heat strain is common on those health workers [9]. To attenuate the heat strain of such occupational workers with heavy labor in hot environments, as well as medical professionals during COVID-19, portable personal cooling systems (PCSs) have been proposed and developed [10,11,12,13,14,15,16,17], including fluid cooling, phase change materials (PCMs) cooling, evaporative cooling, thermoelectric cooling, vacuum desiccant cooling, and radiative cooling. Nevertheless, every cooling approach has limitations [18,19,20,21]. For example, the natural air ventilation might transport ambient heat to the human body if the environmental temperature were greater than the skin temperature; the use of PCMs generally hinders the moisture dissipation from the skin to the ambient environment; the evaporative cooling does not work well in the hot environments with high relative humidity. In order to overcome the inherent limitations of every cooling technique, hybrid cooling systems incorporated with two or more cooling methods in one cooling system were proposed [22,23,24,25]. Webbon et al. [22] found that garments incorporated with the liquid and ventilation cooling could provide better thermal comfort for astronauts as compared to single cooling systems. Wang et al. [23] studied the performance of a liquid cooling garment incorporated with PCM microcapsules, which showed that the inlet temperature, mass flow rate and the volume concentration of PCM microcapsules must be adjusted appropriately to achieve the best cooling performance. Song and Wang [24] integrated PCM packs and ventilation fans into a cooling garment named as a hybrid personal cooling system (HPCS), and found that the HPCS could significantly reduce the heat strain of wearers in a hot, humid environment.

Among existing hybrid personal cooling systems, the combination of PCM packs and ventilation fans has been widely chosen [24,25,26,27]. For HPCSs incorporated with PCMs and ventilation fans, the PCM packs are generally discretely positioned at different body sites. Such a design pattern permits the skin moisture evaporation through the gaps between adjacent PCM packs. Meanwhile, the moisture can also be transported away to the ambient environment by the forced ventilation air. Hence, the relative humidity in the HPCS systems could be reduced significantly and thereby this greatly improves the wetness sensation while wearing the HPCS. In addition, after the PCMs are completely melted, the human body still could be cooled by the ventilation air through convective or/and evaporative heat transfer. Furthermore, even when the ventilation temperature is higher than the skin temperature, the PCM packs could also cool the human body via radiative heat transfer and thereby attenuate the heat stress. Nevertheless, a study by Wan et al. [28] has shown that a prominent portion of the PCM cooling energy was wasted to the ambient environment because the PCM packs could absorb heat from the hot environment. Consequently, the PCM melting process could be accelerated leading to a shorter efficient body cooling duration brought by PCMs.

In order to solve the early melting issue of PCMs in the HPCS, the HPCS was redesigned by adding additional thin EPE (expanded polyethylene) insulation pads in between the PCM packs and the outer clothing layer (referred to as the new HPCS), and its cooling performance influenced by the clothing characteristics was examined by a recent numerical study [29]. Insulation pads were conveniently inserted into discrete pockets that were used for housing the PCM packs. The results showed that the new HPCS could significantly reduce the heat drawn by the PCMs from the hot environment at 36 °C and RH = 59%. Furthermore, the use of high EPE insulation and PCMs with high latent heat and high melting temperature could greatly contribute to improving HPCS’s thermal performance by providing wearers extended cooling duration. Nevertheless, apart from the aforementioned clothing factors, environmental temperature and relative humidity (RH) are also reported to play a significant role in affecting the cooling performance of PCSs [21,30,31,32]. It is anticipated that high environmental temperatures could enhance the melting process of the PCMs and thereby shorten the effective cooling duration of the HPCS. Also, high environmental RHs could suppress the sweat evaporation from the skin surface and thus this weakens the evaporative heat dissipation from the human body. Besides, the moisture from the ambient environment might be condensed on the PCM surface at sufficiently high environmental RHs, which also shortens the PCM melting process and hence reduces the efficient working time of the HPCS. It should be pointed out that results reported in our previous study [29] may not be generalized to other environmental conditions than the chosen condition (i.e., 36 °C, RH = 59%). Therefore, it’s very meaningful to examine the effects of environmental temperature and RH on the cooling performance of the new HPCS so as to guide the energy-efficient application of the HPCS in various environmental conditions.

In this paper, based on the validated numerical model reported in our previous study [26], we performed a numerical parametric study to investigate the effects of environmental temperature and relative humidity (RH) on the cooling performance of the new HPCS. Human physiological responses (e.g., mean skin temperature, local skin temperature, and core temperature) to the new HPCS under different environmental conditions were examined and compared. Besides, the PCM thermal performance was extensively examined in terms of the PCM temperature and PCM heat absorptions from the ambient environment and the human body. In the present parametric study, five levels of environmental temperature under ambient RH = 50% (i.e., 32, 34, 36, 38 and 40 °C) and four levels of environmental RH at 36 °C and 40 °C (i.e., 30, 50, 70 and 90%) were selected for the numerical analysis. This study is of great importance for optimizing the application of the new HPCS to specific hot environments, which is beneficial for reducing heat stress experienced by outdoor workers while working in various hot environments.

## 2. Methodology

### 2.1. Description of the HPCS

**Figure 1:**
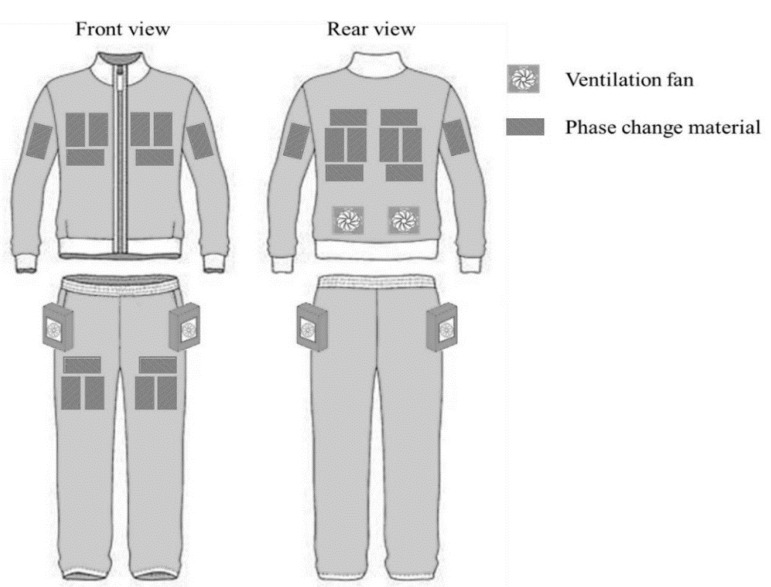
Schematic chart of the new HPCS.

The new HPCS was composed of a long-sleeve jacket and a pair of full-length trousers as indicated in Figure 1. Twenty-four PCM packs with 24 insulation pads were housed in the clothing inner pockets. More specifically, six, eight, four and six PCM packs were located at the chest, upper back, upper arms and thighs, respectively. PCMs must be in close contact with the body so that they could effectively draw heat through conduction and aforementioned body sites were typically contacted with clothing directly. The insulation pads were positioned between the PCM packs and the outer clothing layer. In addition, four ventilation fans with a diameter of 9.8 cm were installed at the lower back and near pelvis region of the HPCS so as to minimize the body movement restriction. The ambient air was blown into the clothing microclimate and the air could only go out of the new HPCS’s microclimate through sleeve openings. Flow rate for each ventilation fan was 0.02 m^3^/s. A detailed air flow direction within clothing microclimate can be found in reference [33].

The material of the underwear and the pocket layer was 100% polyester with the thicknesses of 0.356 and 0.160 mm, respectively. The specific heat capacity and the moisture regain of the polyester fiber were 1340 J·kg^−1^·K^−1^ and 0.4%, respectively [34]. The density, thermal conductivity and water vapor resistance of the underwear were 317 kg·m^−3^, 0.0614 W·m^−1^·K^−1^ and 1.49 m^2^·Pa·W^−1^, respectively. The density, thermal conductivity and water vapor resistance of the pocket layer were 392 kg·m^−3^, 0.0371 W·m^−1^·K^−1^ and 0.56 m^2^·Pa·W^−1^, respectively. The component of the clothing outer layer was 100% cotton (fabric weave: twill) with a thickness of 0.338 mm. The specific heat capacity and moisture regain of the cotton fiber were 1210 J·kg^−1^·K^−1^ and 8.5%, respectively [34]. The density, thermal conductivity and water vapor resistance of the outer clothing layer were 607 kg·m^−3^, 0.0592 W·m^−1^·K^−1^ and 4.0 m^2^·Pa·W^−1^, respectively.

The ingredient of the PCM packs was the sodium sulfate decahydrate (Na_2_SO_4_·10H_2_O) with the latent heat and the melting temperature of 144 kJ/kg and 21 °C, respectively. The density, specific heat capacity and the thermal conductivity of the PCMs were 500 kg·m^−3^, 3600 J·kg^−1^·K^−1^ and 0.6 W·m^−1^·K^−1^, respectively [35]. The length and width of the PCM packs were 120 and 70 mm, respectively, and the total weight of the 24 PCM packs was 1.54 kg. In addition, the material of the insulation pads was expanded polyethylene (EPE) and the thickness of each insulation pad was 5 mm with the corresponding thermal resistance of 0.167 m^2^·K·W^−1^. The density, specific heat capacity and the thermal conductivity of the insulation pads were 20 kg·m^−3^, 1340 J·kg^−1^·K^−1^ and 0.03 W·m^−1^·K^−1^, respectively. The length and width of each insulation pad were the same as those of the PCM packs and the total weight of the 24 insulation pads was 22.0 g.

### 2.2. Numerical Model of the HPCS

**Figure 2:**
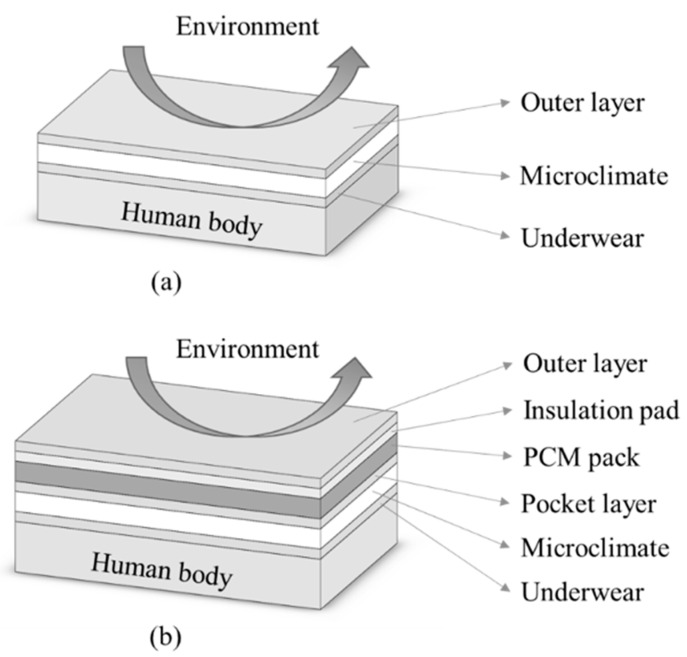
Configurations of clothing-body segments (**a**) without and (**b**) with PCMs and insulation pads considered for the numerical model.

The numerical model describing the heat and mass transfer through the new HPCS was applied to investigate the effects of the environment temperature and RH on the cooling performance of the new HPCS. The thermoregulatory-HPCS model has been validated in the previous study [29]. In this model, the entire body-HPCS system was divided into 5 nude body segments (i.e., head, left foot, right foot, left hand and right hand), 6 clothing-body segments covered with PCMs and insulation layers (i.e., chest, back, left arm, right arm, left thigh and right thigh), and 11 clothing-body segments without the PCMs and insulation layers (i.e., chest, back, pelvis, left shoulder, right shoulder, left arm, right arm, left thigh and right thigh, left leg and right leg). Figure 2 shows the configurations of the clothing-body segments without and with the PCMs and insulation layers considered in the numerical study.

To simplify the problem, the model was approximated to a one-dimensional phenomenon according to the following assumptions [28]:

(1) Local thermal and moisture transports both reach equilibrium instantly in each clothing layer;
(2) The mobile liquid water purely exiting in the underwear layer dispersed into the whole fabric layer throughout the whole segment instantaneously;
(3) The air ventilation by fans is considered as the independent air exchange between the clothing microclimate and the ambient environment for each segment, i.e., no air transport between any adjacent segments. Besides, the HPCS was considered air-tight and air could only go out from the openings at the sleeves (to avoid possible air leakage, an elastic waistband was used at the bottom of the air ventilation jacket and the waist area of air ventilation trousers, the neck area of the jacket was also tightened using zippers).
(4) PCM packs are regarded as a uniform material with a constant thickness, and they closely adhere to the pocket textiles.

In this numerical study, the Tanabe’s 65-node thermoregulation model [36] and a heat and moisture transfer clothing model [34] were coupled to solve this problem. It should be noted that the heat and moisture transfer through the human body and the clothing layers are highly coupled together. In the clothing model, the boundary condition of the underwear surface contacting human skin was obtained from the Tanabe’s thermoregulation model in each time step.

In the Tanabe’s model, each body segment was divided into core, muscle, fat and skin layers in the radial direction. The governing equations of heat transfer in different layers of the body segment are given by:

Core layer (*j* = 1):

(1) $$C\left(i,1\right)\frac{dT\left(i,1\right)}{dt}=Q\left(i,1\right)-B\left(i,1\right)-D\left(i,1\right)-RES\left(i,1\right)$$

Muscle layer (*j* = 2):

(2) $$C\left(i,2\right)\frac{dT\left(i,2\right)}{dt}=Q\left(i,2\right)-B\left(i,2\right)+D\left(i,1\right)-D\left(i,2\right)$$

Fat layer (*j* = 3):

(3) $$C\left(i,3\right)\frac{dT\left(i,3\right)}{dt}=Q\left(i,3\right)-B\left(i,3\right)+D\left(i,2\right)-D\left(i,3\right)$$

Skin layer (*j* = 4):

(4) $$C\left(i,4\right)\frac{dT\left(i,4\right)}{dt}=Q\left(i,4\right)-B\left(i,4\right)+D\left(i,3\right)-Q_{t}\left(i,4\right)-E\left(i,4\right)$$

where, *i* and *j* indicate the segment number and the body layer number, respectively; *C* is heat capacity; *T* is temperature; *t* is time; *Q* is the heat production rate of the body; *B* is the heat exchange with the blood compartment; *D* is the conductive heat transfer between adjacent layers; *RES* is the respiration heat loss, which is only applicable for the chest segment; *Q_t_* is the dry heat exchange (i.e., convective and radiant) between the skin and the environment; *E* is the evaporative heat loss from the skin surface. The dry heat exchange, *Q_t_* (*i*, 4), could be obtained by the temperature difference between the skin and the microclimate/environment, while the evaporative heat loss from the skin surface, *E*(*i*, 4), could be calculated based on the vapor pressure difference between the skin surface and the microclimate/environment.

**Figure 3:**
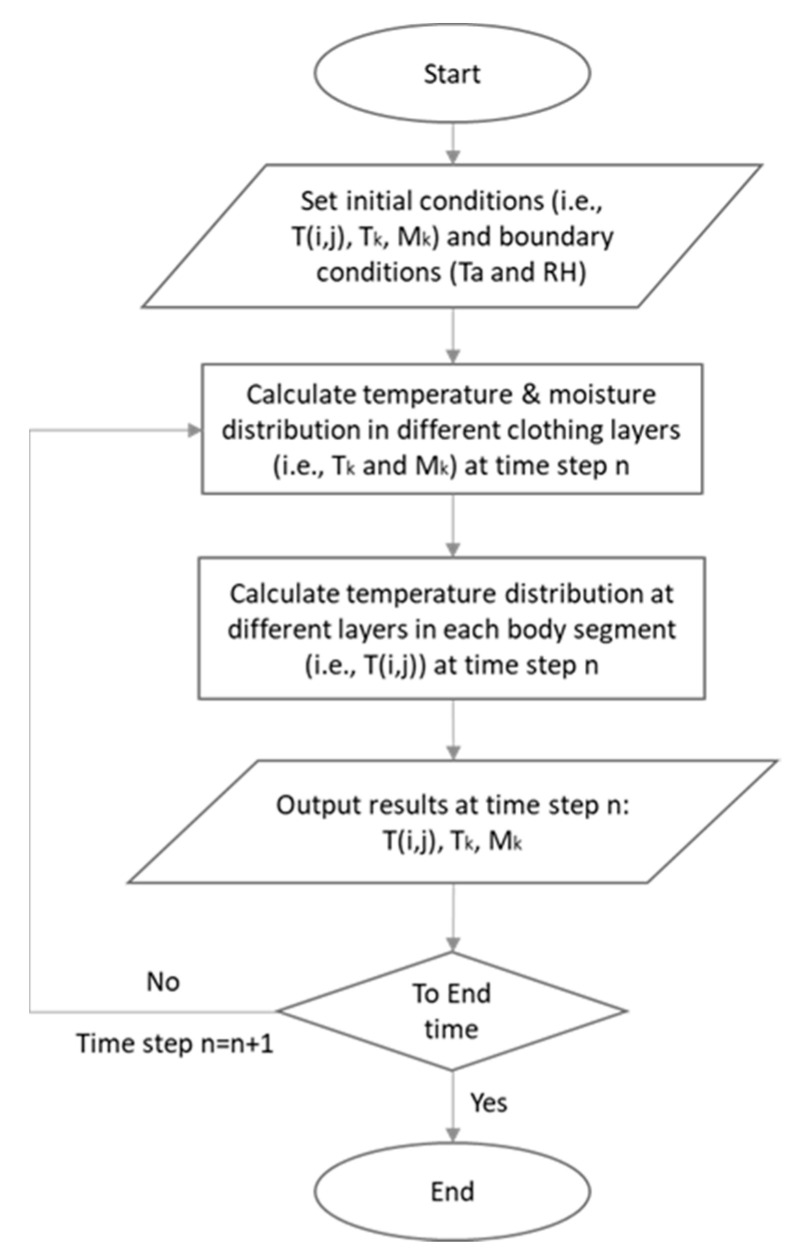
Flow chart showing the operation sequence of the new HPCS model.

In the HPCS model, heat transfer takes place through all clothing layers from the underwear layer to the outer clothing layer, whereas the moisture transport only occurs in fabric layers and the ventilation air layer (i.e., microclimate). Governing equations of the heat and moisture transfer through the HPCS system are given by:

(5) $$\rho_{k}V_{k}C_{p,k}\frac{dT_{k}}{dt}=q_{c,k-1}-q_{c,k}+q_{l,k}-q_{v,k}$$

(6) $$\frac{dM_{k}}{dt}=D_{k-1}-D_{k}+M_{v,k}+M_{s,k}$$

where, *k* denotes the clothing layer; *t* is time; *ρ_k_* is density; *C_p,k_* is specific heat capacity; *V_k_* is the volume of the discretized clothing layer *k*; *T_k_* is temperature; *M_k_* is moisture mass; *q*_*c*,*k*−1_ and *q_c,k_* are conductive/convective heat flux from layer *k* − 1 to layer *k* and from layer *k* to layer *k* + 1, respectively; *D*_*k*−1_ and *D_k_* are moisture mass flux by diffusion/convection from layer *k −* 1 to layer *k*, and from layer *k* to layer *k +* 1, respectively; *q_l,k_* is heat released by condensation/absorption of water vapor in fabric layers; *q_v,k_* and *M_v,k_* are heat and moisture transfer by air ventilation, respectively; *M_s,k_* is the sweat captured by the innermost layer (i.e., underwear) of the clothing system, which is mainly decided by the sweat accumulation on the skin surface. The sweat accumulation on the skin surface (i.e., *M_sa_*) is calculated by:

(7) $$\frac{dM_{sa}}{dt}=\frac{P_{sat}\left(T_{skin}\right)-P_{skin}}{\lambda R_{e,skin}}+{\dot{m}}_{rsw}$$

where, *T_skin_* is the skin temperature, i.e., *T_skin_* = *T*(*i*, 4); *P_skin_* is the partial water vapor pressure at the skin surface; *P_skin_* = *P*(*i*, 4); *P_sat_*(*T_skin_*) is the saturated water vapor pressure at the skin surface; *R_e,skin_* is the water vapor resistance of the skin (for a hydrated person, *R_e,skin_* = 330 m^2^·Pa/W); ${\dot{m}}_{rsw}$ is the sweat secretion rate, which is calculated based on the work of Tanabe et al. [36]. It should be noted that the sweat accumulation on the skin surface should never exceed 35 g/m^2^. If not, the sweat will be absorbed by the underwear and the absorption rate is referred to as *M_s,k_*. Also, if the body parts are not covered with clothing (e.g., hands and the head), the sweat drips off the skin accordingly. For PCM layers, the so-called apparent heat capacity method [37,38] is applied to solve the heat transfer equations. For detailed information on the coupling of Equations (5) and (6) with the Tanabe human thermoregulatory model, please refer to references [34,36]. Governing equations were discretized by the finite difference method (FDM) using an explicit scheme. PCM layers, fabric layers and insulation layers include 50, 10 and 6 discrete nodes, respectively. The time step was set to 0.05 s. The simulation code was programmed through MATLAB to couple the Tanabe’s thermoregulatory model with the new HPCS clothing model. The operation sequence of the numerical HPCS-thermoregulatory model is shown in a flow chart displayed in Figure 3.

### 2.3. Simulation Parameters

The numerical simulation investigated scenarios involving a 70-min moderate-intensity work (metabolic rate: 4.2 METs) followed by a 20-min rest period (metabolic rate: 1.2 METs). The initial PCM temperature was set around 12 °C (which was taken from the reference [29]). A parametric study was carried out to explore the effects of the environmental temperature and RH on the thermal performance of the new HPCS. The environmental RH was set to 50% to study the effect of the environmental temperature on the HPCS’s cooling performance. Two representative hot environments were chosen to study the effect of the environmental RH on the cooling performance of the new HPCS: a moderately hot environment (i.e., 36 °C) and an extremely hot environment (i.e., 40 °C). Table 1 gives the variables of the environmental temperature and RH investigated in the numerical parametric study. It could be seen from Table 1 that five levels of environmental temperature under ambient RH = 50% (i.e., 32, 34, 36, 38 and 40 °C) and four levels of environmental RH at 36 and 40 °C (i.e., 30, 50, 70 and 90%) were selected for the numerical analysis. These conditions were used to simulate commonly reported weather conditions in subtropical regions (e.g., Shanghai and Hong Kong) during summer. The air velocity was set to 0.15 m/s.

## 3. Results and Discussion

In the following sections, the chest segment covered with/without PCMs and insulation pads is chosen as the representative body segment to study the effects of the environmental temperature and RH on the thermal performance of the new HPCS.

### 3.1. Effect of Environmental Temperatures

**Figure 4:**
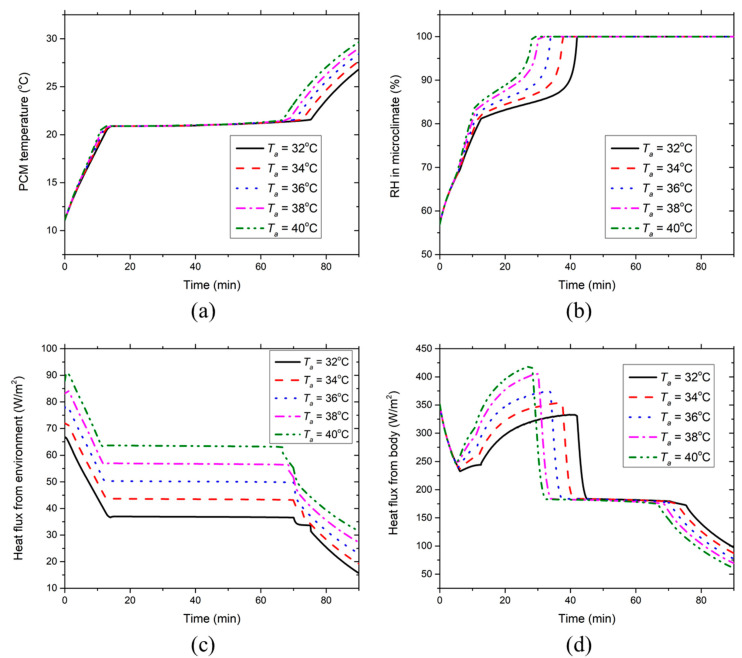
Effect of environmental temperature on the time course of (**a**) PCM temperature, (**b**) clothing microclimate RH, (**c**) PCM heat absorption from the environment and (**d**) PCM heat absorption from the body at the chest segment covered with PCMs and insulation pads at RH = 50%.

Figure 4 presents the effect of the environmental temperature on the development of the PCM temperature, clothing microclimate RH, heat fluxes absorbed by the PCMs from the environment and the body at the chest segment covered with PCMs and insulation pads at RH = 50%. Figure 4a indicates that the PCM temperature increases linearly during the initial 10 min due to the sensible heat absorption of the low-temperature PCMs. Subsequently, the PCM temperature maintains constant at 21 °C (i.e., the melting temperature) during the melting process. After the PCMs are melted, the PCM temperature starts to rise again because of the sensible heat absorption of liquid PCMs from the body and the environment. As the environmental temperature increases, the PCM melting time-duration reduces due to the larger amount of heat absorption from the environment at higher ambient temperatures (see Figure 4c). It is evident that the environmental temperature has a minor effect on the PCM temperature before the PCMs are melted. Figure 4b indicates that the clothing microclimate RH increases promptly during the initial 10 min of the simulation. The initial great increment of microclimate RH is mainly due to the continuous sweat production and moisture condensation on the PCM inner surface. With the further increase in the microclimate RH, the sweat evaporation from the skin tends to be suppressed, whereas the moisture in the microclimate of the HPCS increasingly condenses on the inner surface of the PCMs due to the low PCM temperature and high microclimate RH. Hence, the microclimate RH increases gradually in the following phase after about the 10th min. When the equilibrium between the moisture condensation and evaporation is reached on the inner surface of the PCM packs, the moisture in the clothing microclimate could not be further condensed on the surface of PCM packs. In contrast, the sweat on the skin surface continues evaporating. The microclimate RH will rise dramatically to 100% in about 25 to 42 min at the five selected temperatures. It is evident that the higher the environmental temperatures, the less time required for the microclimate RH to reach 100%. This is because the high environmental temperature induces a high saturated vapor pressure in the clothing microclimate and thus the sweat evaporation from the skin will be enhanced. Once the microclimate RH reaches 100%, it will last until the end of the simulation regardless of the environmental temperatures.

**Figure 5:**
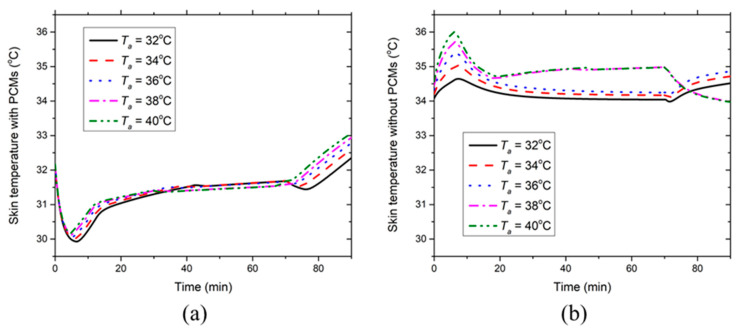
Effect of environmental temperature on the time course of chest skin temperatures (**a**) with and (**b**) without PCMs and insulation layers at RH = 50%.

Figure 4c shows that the heat flux absorbed by the PCMs from the environment decreases until around the 10th minute due to the increasing PCM temperature. Afterward, the heat flux reaches a lower steady level induced by the constant melting temperature of the PCMs. After the PCMs are melted, the heat flux from the environment starts to decrease again, which is caused by the corresponding PCM temperature increase as indicated in Figure 4a. The heat absorption by PCMs from the environment increases with the increasing environmental temperature because the high environmental temperature leads to a large temperature gradient between the PCMs and the environment. The heat flux absorbed by the PCMs from the body exhibits a significant drop during the initial five minutes (see Figure 4d). This is caused by the PCM temperature growth (see Figure 4a), as well as the local skin temperature decrease (see Figure 5a). Afterward, the heat flux increases greatly to the maximum value, which is probably induced by the increasing local skin temperature and also by the increasing moisture condensation on the PCM surface due to the growing microclimate RH. During this period (i.e., the 10th to 30th minute), the heat flux drawn from the body increases with the increasing environmental temperature, which is because higher environmental temperatures result in higher microclimate RHs and thereby enhance the moisture condensation heat release on the PCM surface. After the microclimate RH reaches 100%, the moisture in the clothing microclimate is not able to condense on the PCM surface, leading to the absence of the heat released by moisture condensation. As a result, the heat flux from the body steps down from the highest value to a lower level (during the PCM melting process) once the microclimate RH rises to 100% (see Figure 4b,d). After PCMs are melted, the heat flux from the body starts to sharply decrease because of the temperature increase in the liquid PCMs and, meanwhile, it decreases with the increasing environmental temperature because high environmental temperatures correspond to high PCM temperatures (see Figure 4a) leading to small temperature gradients between the body and PCMs.

Figure 5 presents the effect of environmental temperature on the development of the chest skin temperature with and without PCMs and insulation layers at RH = 50%. Figure 5a indicates that the local chest skin temperature covered with PCMs and insulation pads decreases until the fifth minute due to the low initial PCM temperature. Then, the skin temperature increases to a relatively steady period as the PCM temperature increases to the melting temperature. The environmental temperature has a limited effect on the chest temperature covered with PCMs during the 70-min moderate-intensity work. After the PCMs are melted, the skin temperature starts to rise because the increasing PCM temperature reduces the heat dissipation from the skin. It should be noted that the skin temperature has a slight drop at the 70th minute at 32 °C, which is because the heat production declines due to the termination of the moderate-intensity work but the PCMs are still under the melting process. In contrast, Figure 5b shows that the local chest skin temperature without PCMs and insulation layers increases in the initial five minutes because of the large heat production resulting from the moderate-intensity work. Meanwhile, during this period, the high environmental temperature corresponds to the high local skin temperature. Afterward, the skin temperature decreases to a relatively lower level because the increasing skin temperature induces large sweat generation and thereby intensive evaporative heat dissipation from the skin. When the ambient temperature is ≤36 °C, it has a minor effect on the skin temperature and the chest temperature increases after the 70th minute. This is because the termination of the moderate-intensity work induces low sweat generation leading to smaller evaporative heat dissipation from the skin. However, when the environmental temperature is ≥38 °C, the local chest skin temperature is much higher than that in the aforementioned cases (≤36 °C) and it decreases after the 70th minute. At sufficiently high environmental temperatures, the ventilation air could transport a lot of heat to the skin leading to a high sweat generation that cannot be completely evaporated. In these cases, the skin is covered with sweat film and the evaporative heat dissipation reaches the maximum value, which yet cannot remove the generated body heat. Consequently, the local skin temperature increases due to the large heat transfer from the ventilation air to the skin. After the termination of the moderate-intensity work, the metabolic heat generation rate decreases, whereas the evaporative heat dissipation is still intensive due to the existing sweat film. Hence, the skin temperature without PCMs and insulation layers declines after the 70th minute at sufficiently high environmental temperatures (e.g., ≥38 °C).

**Figure 6:**
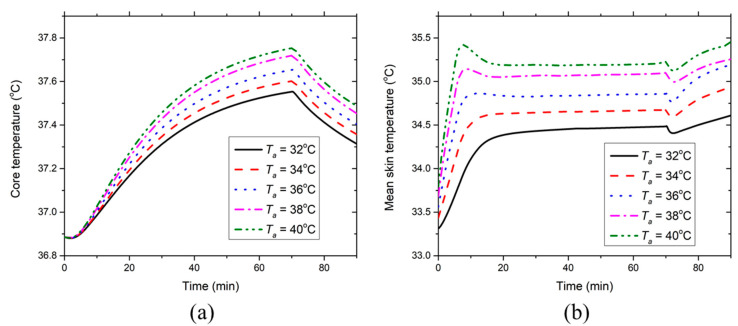
Effect of environmental temperature on the time course of (**a**) core temperature and (**b**) mean skin temperature at RH = 50%.

Figure 6 displays the effect of the environmental temperature on the development of the core temperature and the mean skin temperature at RH = 50%. It can be seen from Figure 6a that the core temperature increases during the first 70 min and then it decreases because of the termination of work. Obviously, the core temperature increases with the increasing environmental temperature. This is because high environmental temperature conditions suppress body heat dissipation to the ambient environment. If the environmental temperature is larger than the skin temperature (e.g., 38 and 40 °C), the ventilation air could even transport ambient heat to the body through convection, which also contributes to high core temperature rises. In such situations, severe heat strain and heat-related illnesses could be induced. With regard to the mean skin temperature, it increases continuously during the initial 10 min and afterward, it is maintained at a plateaued level between 34.3 and 35.5 °C until the 70th minute (see Figure 6b). The initial 10-min skin temperature rise demonstrates that the skin temperature increment of all the non-PCM covered segments outweighs the temperature decrement of all the PCM-covered segments during this period. After the initial 10 min, both the skin temperatures with and without PCMs reach the steady levels as shown in Figure 5. At the 70th minute, the mean skin temperature has a slight drop and then starts to increase. The temperature drop is probably caused by the sudden heat production decrease after the termination of the work. The following temperature increase is induced by the PCM temperature increase in the PCM covered segments and also by the low sweat evaporative heat dissipation in the non-PCM covered segments. Moreover, it is obvious that the mean skin temperature increases with the increasing environmental temperature.

### 3.2. Effect of Relative Humidity in Moderately Hot Environments (36 °C)

**Figure 7:**
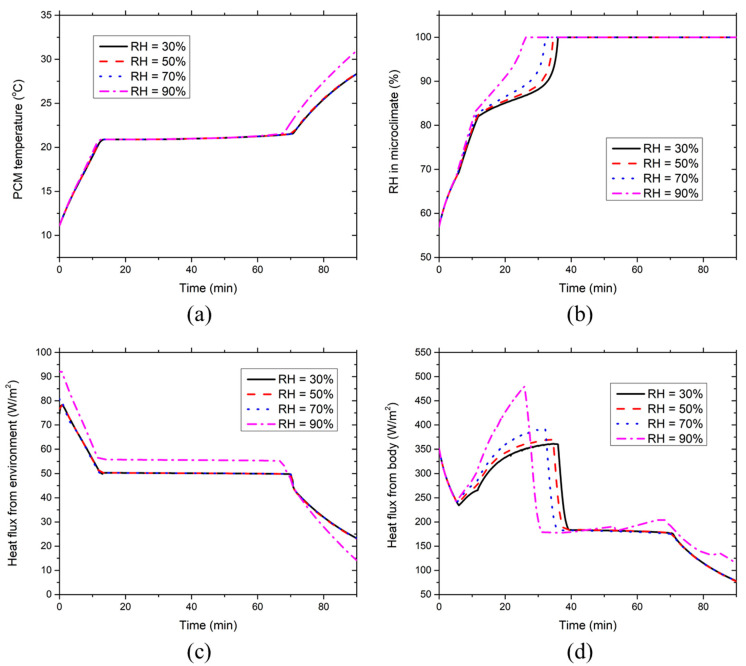
Effect of environmental RH on the time course of (**a**) PCM temperature, (**b**) clothing microclimate RH, (**c**) PCM heat absorption from the environment and (**d**) PCM heat absorption from the body at the chest segment covered with PCMs and insulation pads at the ambient temperature of 36 °C.

Figure 7 demonstrates the effect of the environmental RH on the development of the PCM temperature, clothing microclimate RH, heat fluxes absorbed by PCMs from the environment and the body at the chest segment covered with PCMs and insulation pads at 36 °C. Figure 7a shows the PCM temperature increases from the initial temperature to the melting temperature (21 °C) in the initial 10 min. Then, the PCM temperature maintains constant during the PCMs’ melting process. After the PCMs are melted, the PCM temperature continues to rise due to the sensible heat absorption of liquid PCMs. The environmental RH has little effect on the PCM temperature when the environmental RH ≤ 70%. In contrast, the melting duration of the PCMs is shortened by about five minutes at RH = 90%, which is because there is too much heat absorption by moisture condensation on the PCM surface at high RH. Hence, the PCM temperature at RH = 90% is the highest throughout the 65–90th minute. Figure 7b describes that the clothing microclimate RH increases rapidly until about the 10th minute and then it undergoes a gradual growing phase, after which the microclimate RH rises dramatically to 100%. The reason is similar to that described in Section 3.1. The environmental RH has a minor effect on the microclimate RH when RH ≤ 70%, whereas the microclimate RH increases rapidly to 100% during the gradual increase phase at RH = 90%. At the sufficiently high environmental RH, the ventilation air contains too much moisture and thus the condensation and evaporation on the PCM surface reach equilibrium at a much earlier time. As a result, the microclimate RH quickly increases to the saturated level at RH = 90%.

**Figure 8:**
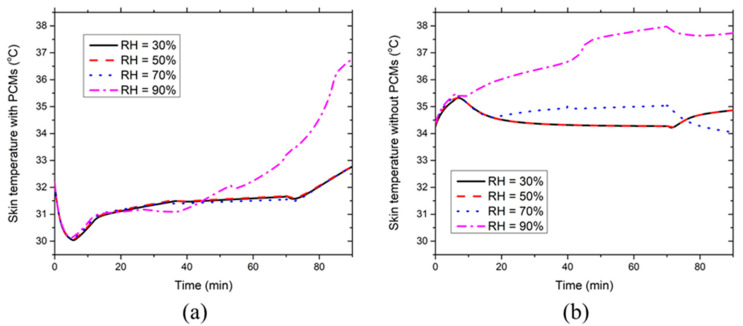
Effect of environmental RH on the time course of chest skin temperatures (**a**) with and (**b**) without PCMs and insulation layers at the ambient temperature of 36 °C.

Figure 7c shows that the heat flux absorbed by the PCMs from the environment declines to a lower stable level during the first 10 min, which corresponds to the initial PCM temperature increment as indicated in Figure 7a. Afterward, the stable level maintains during the entire PCM melting process. Once the PCMs are melted, the heat flux starts to decrease again because the increasing PCM temperature reduces the temperature gradient between the PCMs and the environment. The environmental RH has little effect on the heat absorption by the PCMs from the hot environment when RH ≤ 70%. However, at RH = 90%, the heat absorption from the environment is larger than the other cases (i.e., RH ≤ 70%) before the PCMs are melted, which is caused by more moisture condensation on the outer surface of the PCMs. In contrast, after the PCMs are melted, the heat flux at RH = 90% becomes smaller than those at RH ≤ 70%, which is induced by the high PCM temperature as indicated in Figure 7a. Figure 7d illustrates that the heat flux absorbed by the PCMs from the human body decreases until about the fifth minute and then increases to the highest value. Subsequently, the heat flux drops dramatically to a lower stable level, after which it starts to decrease almost linearly. The reason is similar to that demonstrated in Section 3.1. The environmental RH has a limited effect on the heat flux drawn by the PCMs from the body when RH ≤ 70%. As for the RH = 90% case, the heat flux increases rapidly from the local lowest point at around the fifth minute to the highest value, because the corresponding high microclimate RH induces intensive moisture condensation heat release on the PCM surface. It is interesting to note that the heat flux at RH = 90% starts to increase at around the 45th minute during the PCM melting process, which is caused by the increase in the local skin temperature covered with PCMs as shown in the following discussion (see Figure 8a).

Figure 8 illustrates the effect of the environmental RH on the development of the chest skin temperature with and without PCMs and insulation layers at 36 °C. Figure 8a indicates that the local chest skin temperature covered with PCMs first decreases during the initial five minutes and then increases to a relatively steady level. At RH ≤ 70%, the skin temperature starts to rise after the PCMs are melted and RH has little effect on the local chest skin temperature covered with PCMs. In contrast, the skin temperature rise appears at around the 45th minute during the melting process of the PCMs when RH reaches 90%. This is because high RH significantly suppresses the evaporative heat dissipation from the skin, whereas the heat production rate is still intensive during the moderate-intensity work. Consequently, the skin temperature starts to increase rapidly during the PCM melting process, which corresponds to the heat flux rise at around the 45th minute as shown in Figure 7d. Figure 8b shows that the local chest skin temperature without PCMs increases until about the fifth minute. Afterward, the skin temperature decreases to a relatively steady level at RH ≤ 70% due to the evaporative heat dissipation from the skin. As for the RH = 90% case, the local skin temperature continues to increase rapidly until the 70th minute. In this case, the evaporative heat dissipation rapidly reaches the maximum value, which is not yet sufficient to take away the body heat generated. In addition, the skin temperature at RH = 70% is larger than that at RH = 30/50% during the relatively steady stage. This is because the evaporative heat dissipation at RH = 70% has already reached the maximum value at about the 20th minute and thereby, the skin temperature has to maintain a higher level to reduce the convective heat transfer from the ventilation air to the skin. Noticeably, the skin temperature at RH ≤ 50% starts to increase from the 70th minute because the termination of the moderate-intensity work reduces the sweat generation weakening the evaporative heat dissipation. In contrast, the skin temperature at RH ≥ 70% decreases from the 70th minute. This is because the evaporative heat dissipation is still intensive due to the existing sweat film on the skin.

**Figure 9:**
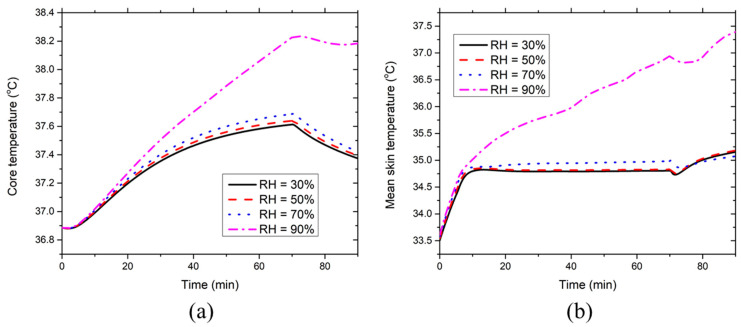
Effect of environmental RH on the time course of (**a**) core temperature and (**b**) mean skin temperature at the ambient temperature of 36 °C.

Figure 9 presents the effect of the environmental RH on the development of the core temperature and mean skin temperatures at 36 °C. Figure 9a indicates that the environmental RH has a minor effect on the core temperature when RH ≤ 70%. However, the core temperature at RH = 90% increases significantly (even higher than 38 °C), which is induced by the suppression of the evaporative heat dissipation due to the high moisture content in the ventilation air. Similarly, Figure 9b denotes that the environmental RH has a limited effect on the mean skin temperature at RH ≤ 70%, while the mean skin temperature increases greatly at RH = 90%. Therefore, the new HPCS is not suggested for use in the moderately hot environment (36 °C) at RH = 90%.

### 3.3. Effect of Relative Humidity in Extremely Hot Environments (40 °C)

**Figure 10:**
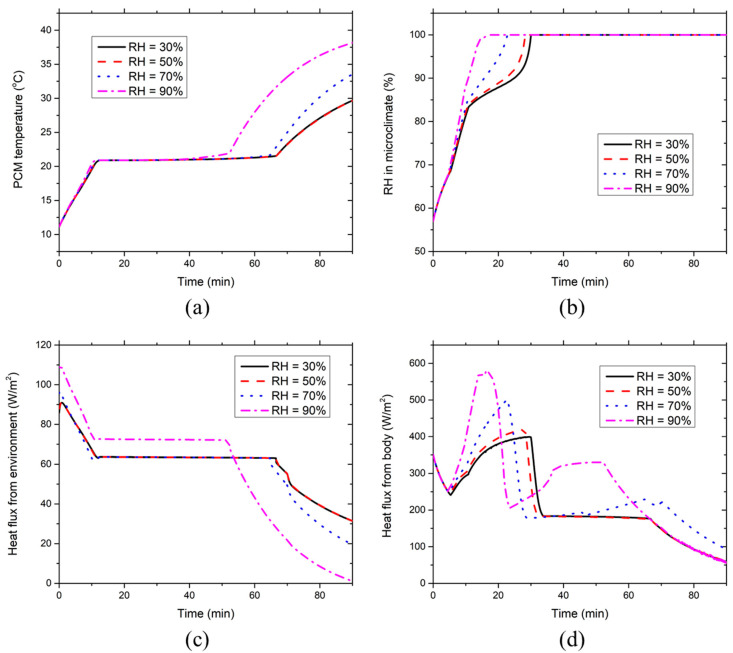
Effect of environmental RH on the time course of (**a**) PCM temperature, (**b**) clothing microclimate RH, (**c**) PCM heat absorption from the environment and (**d**) PCM heat absorption from the body at the chest segment covered with PCMs and insulation pads at the ambient temperature of 40 °C.

Figure 10 demonstrates the effect of the environmental RH on the development of the PCM temperature, clothing microclimate RH, heat fluxes absorbed by PCMs from the environment and the body at the chest segment covered with PCMs and insulation pads at 40 °C. Figure 10a indicates that the environmental RH has little effect on the PCM temperature when RH ≤ 50%. As for RH ≥ 70% cases, the melting period of the PCMs decreases with the increasing RH and, the higher RH corresponds to the higher PCM temperature after the PCMs are melted. Figure 10b denotes that the environmental RH has a minor effect on the clothing microclimate RH when the RH ≤ 50%. In contrast, as for RH ≥ 70% cases, the microclimate RHs during the gradual increment are much larger than those at RH ≤ 50%, and the microclimate RH increases with the increasing RH. It should be noted that clothing microclimate RH increases rapidly to 100% without showing the gradual increment at RH = 90% because of the high ambient moisture content and pronounced sweat generation in the extremely hot, humid environment.

**Figure 11:**
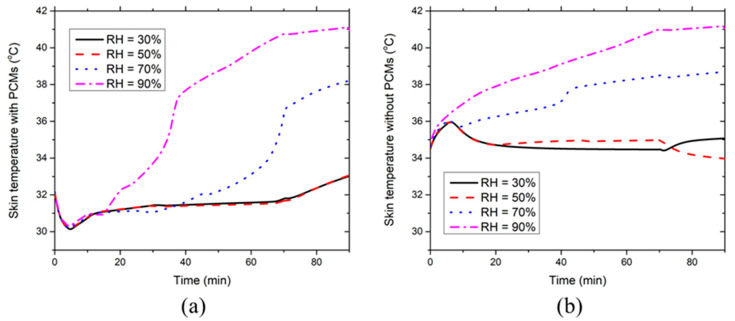
Effect of environmental RH on the time course of chest skin temperatures (**a**) with and (**b**) without PCMs and insulation layers at the ambient temperature of 40 °C.

Figure 10c shows that the environmental RH has little effect on the heat flux drawn by the PCMs from the environment when RH ≤ 50%. In contrast, at RH = 90%, heat fluxes during the initial decreasing phase and the following steady period are higher than those in the other cases because of the intensive moisture condensation that took place on the PCM packs’ outer surface. Moreover, the heat flux at RH ≥ 70% decreases with the increasing environmental RH after the PCMs are totally melted, which is caused by the high PCM temperature at high environmental RH as indicated in Figure 10a. Figure 10d shows that the environmental RH has a limited effect on the heat absorption by the PCMs from the body at RH ≤ 50%. However, as for RH ≥ 70% cases, the highest value of the heat flux increases significantly with the increase in the environmental RH. This is because the high microclimate RH (see Figure 10b) enhances the moisture condensation heat release on the PCM surface. Meanwhile, after the step-down process of the heat flux at microclimate RH = 100%, heat fluxes at RH ≥ 70% start to increase during the PCM melting process and the time point at which the skin temperature starts to increase would be earlier at higher RH conditions. This is induced by the increasing local skin temperature covered with PCMs as shown in the following discussion (see Figure 11a).

Figure 11 illustrates the effect of the environmental RH on the development of the chest skin temperature with and without PCMs and insulation layers at 40 °C. Figure 11a indicates that the environmental RH has little effect on the local chest skin temperature covered with PCMs when the RH ≤ 50%. In contrast, when the ambient RH increases to ≥70%, heat gain significantly outweighs the heat loss and the chest temperatures increase greatly even during the PCM melting process. The time point at which chest temperature starts to increase appears earlier with the increasing environmental RH. This is mainly because high environmental RH suppresses the evaporative heat dissipation from the skin to the environment, whereas the ventilation air intensively transports heat to the skin due to the extremely high environmental temperature. Further, it can be seen from Figure 11b that the local chest skin temperature without PCMs increases continuously throughout the entire 90-min simulation under RH ≥ 70% conditions. It is evident that the new HPCS is no longer able to offer sufficient body cooling under such extreme environmental conditions. Meanwhile, higher environmental RHs result in greater chest temperatures.

**Figure 12:**
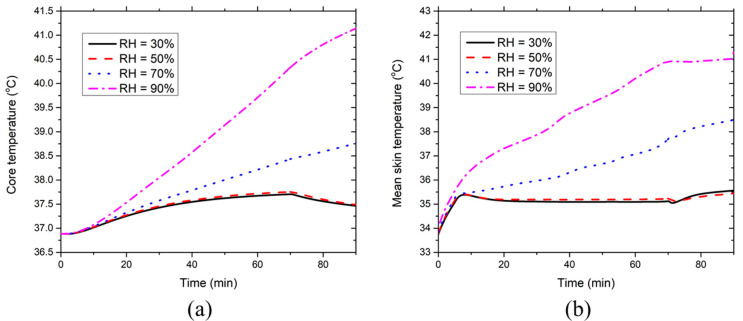
Effect of environmental RH on the time course of (**a**) core temperature and (**b**) mean skin temperature at the ambient temperature of 40 °C.

Figure 12 displays the effect of the environmental RH on the development of the core temperature and the mean skin temperature at 40 °C. The environmental RH has a limited effect on the core temperature and mean skin temperature when RH ≤ 50%. In contrast, both the core temperature and mean skin temperature increase significantly when RH ≥ 70% and the greater environmental RHs correspond to the higher core and mean skin temperatures. This has indicated that the new HPCS could no longer be able to provide sufficient body cooling under such extreme environmental conditions. Compared with the results shown in the moderately hot environment in Section 3.2, the environmental RH to induce a core temperature of 37.5 °C decreases from 70 to 50% when the ambient temperature increases from 36 to 40 °C. Therefore, the new HPCS should be improved by adopting higher insulation EPE pads and PCMs with higher latent heat and low melting temperature based on our previous study [29] so as to make it applicable for the outdoor workers while working in the studied extremely hot humid conditions.

Table 2 presents the maximal core temperature and mean skin temperature observed in the various studied simulation environments. It could be seen from Table 2 that the maximal core temperature reached over 38.0 °C at RH = 90% and temperatures of 36 to 40 °C. A core temperature of 38.0 °C has been widely adopted as the threshold limit to protect workers by the WHO (World Health Organization) and ACGIH (American Conference of Governmental Industrial Hygienists) [39,40]. Hence, the workers are required to take rest under such harsh working conditions even wearing the new HPCS. The maximal core temperature registered in the remaining studied simulation conditions did not exceed the aforementioned temperature threshold limit and thus, the new HPCS could be worn to protect the workers while working in those environmental conditions.

## 4. Conclusions

This numerical parametric study showed that both the core temperature and mean skin temperature increase with the increasing environmental temperature, which means high environmental temperature weakens the thermal performance of the new HPCS. In the moderately hot environment (36 °C), the environmental RH has a limited effect on the thermal performance of the new HPCS under RH ≤ 70%. Hence, the new HPCS could effectively protect workers against heat stress at any humidity level chosen for the study at a 36 °C temperature and RH < 70%. In contrast, in the extremely hot environment (40 °C), the environmental RH has a minor effect on the thermal performance of the new HPCS under RH ≤ 50%, whereas both the core temperature and the mean skin temperature increase significantly under RH ≥ 70% conditions. The environmental RH to induce a core temperature of 37.5 °C declines from 70 to 50% when the ambient temperature rises from 36 to 40 °C. Moreover, workers could still develop hyperthermia (*T_core_* = 38.5 °C) at 40 °C and 90% RH conditions in less than 40 min even with the new HPCS. Hence, workers should reduce their work duration under such harsh conditions for safety concerns. Lastly, in working conditions with extremely high temperatures/RHs (e.g., 40 °C and 90% RH), the new HPCS should be further improved by adjusting the PCM properties and/or the EPE insulation levels to optimize the thermal performance of the new HPCS. Future experimental studies should be carried out to optimize the HPCS in various hot environments.

## Figures and Tables

**Table 1:** Environmental temperatures and RHs considered in the parametric study.

Variables	Values
Environmental temperature (°C)	32, 34, 36, 38, 40
Environmental RH at 36 °C (%)	30, 50, 70, 90
Environmental RH at 40 °C (%)	30, 50, 70, 90

**Table 2:** Maximal core temperature and mean skin temperature observed in various studied simulation scenarios.

Simulated Temperature (°C)	Maximal Core Temperature (°C)				Maximal Mean Skin Temperature (°C)			
	RH = 30%	RH = 50%	RH = 70%	RH = 90%	RH = 30%	RH = 50%	RH = 70%	RH = 90%
32	-	-	37.57	-	-	34.64	-	-
34	-	-	37.62	-	-	34.92	-	-
36	37.58	37.62	37.70	38.28	35.24	35.24	35.21	37.48
38	-	-	37.75	-	-	35.31	-	-
40	37.73	37.74	37.77	41.20	35.75	35.48	38.58	41.22

Note: -—not applicable.
